# Synthesis and Properties of N-Bromosuccinimide Derivatives of Ammonium Dialkyl/Alkylene Dithiophosphates

**DOI:** 10.1155/2007/24742

**Published:** 2007-02-05

**Authors:** Neelam Harkut, Padam N. Nagar

**Affiliations:** Department of Chemistry, University of Rajasthan, Jaipur 302004, India

## Abstract

Reaction of N-bromosuccinimide with ammonium salt of alkylene (dialkyl) dithiophosphates, [OGOPS_2_NH_4_; G=−CMe_2_CMe_2_−CH_2_CMe_2_CH_2_−, CMe_2_CH_2_CHMe_2_−, CH_2_CH_2_CHMe_2_−; (RO)_2_PS_2_NH_4_; R=C_2_H_5_, n−C_3_H_7_, i−C_3_H_7_], in 1 : 1 molor ratio in refluxing benzene solution yields red/brown-colored sticky liquids. These newly synthesized complexes have been characterised by physicochemical and spectroscopic techniques (MW, IR, NMR [^1^H and ^31^P]). The computational semiempirical calculations (MOPAC6P/c) have also been studied for these complexes. On the basis of the above studies, the formation of P−S−N chemical linkage has been established.

## 1. INTRODUCTION

A lot of work had been reported on organic, metal, and organometal derivatives of O, O′ alkylene/dialkyl dithiophosphates from our laboratories [1–10] during the last decade. In continuation to our earlier investigations on synthesis and properties of phenyl acetyl, p-methyl benzoyl [11], 2-alkylene dialkyl dithiophosphato-2-oxo-1,3,2-dioxa phosphorinane [12], phosphorus trichloride [13], and thio phosphoryl chloride [14] derivatives of alkylene dithiophosphates, it was considered of interest to extend the investigations on the derivatives of the above ligands with N-bromosuccinimide.

## 2. RESULTS AND DISCUSSION

**Scheme 1:**
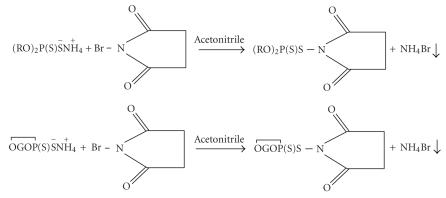


Reaction of N-bromosuccinimide with ammonium dialkyl (alkylene) dithiophosphates has been carried out in the presence of anhydrous acetonitrile which proceeds to completion within in 6-7 h (refluxing) with the precipitation of ammonium bromide as shown in Scheme 1.

**Figure 1:**
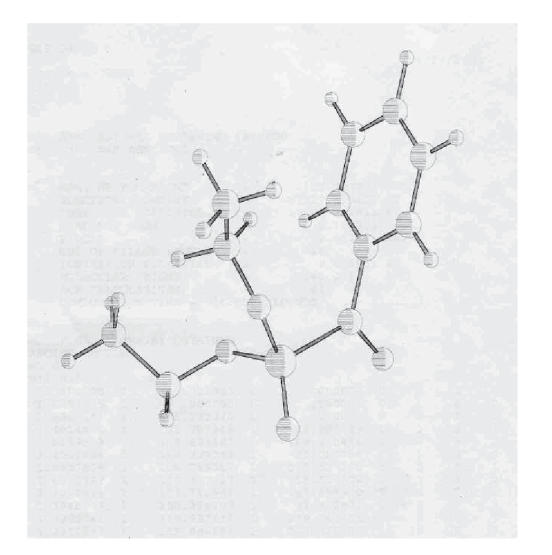
1-[(diethoxy phosphorothioyl)thio] pyrrolidine-2,5-dione.

The derivatives shown in Scheme 1 are reddish- or brown-colored sticky liquids. They are nonvolatile even under reduced pressure and are miscible with common organic solvents. On heating, these compounds tend to decompose. Besides, for these compounds are reddish-colored sticky liquids, it could not be possible to have a single crystal X-ray data for correlation. The computational semiempirical calculations (Mopac6p/c) for one representative complex {1-[(diethoxy phosphorothioyl)thio] pyrrolidine-2,5-dione} have been studied and the data regarding the structure of molecule has been tabulated, (see Table 1 and Figure 1). The molecular weight data corresponds with the results obtained. The molecular data indicates a large deviation in the tetrahedral-bond angle values for almost all the atoms. It also indicates a good stability of the molecule (ΔH_*f*_ = −178.8 K·Cal). The five membered ring at nitrogen atom seems to be puckered. A distorted tetrahedral structure around phosphorus atom has been proposed. These computer-based calculations/data are also in good agreement with the analytical and spectroscopic (IR, PMR, ^31^P NMR) data obtained for these compounds.

### 2.1. Spectral studies

#### 2.1.1. IR spectra

The IR spectra of these derivatives show the following characteristic changes, (see Table 2).

1. The *ν*C=O absorption band is present in the region 1700–1680 cm^−1^. No change in its position has been observed.
2. The *ν*P−S−N absorption band present in the region 1270–1250 cm^−1^ has been shifted towards lower-wave number (20–30 cm^−1^) for six membered (dioxaphosphorinane) ring derivatives.
3. A strong absorption band present in the regions 1125–1020 cm^−1^ and 905–865 cm^−1^ has been observed for *ν*(P)−OC and *ν*P−O(C) stretching vibrations, respectively.
4. The *ν*N−Br absorption band present in the region 912–866 cm^−1^ has been disappeared, which supports the formation of >P−S−N chemical linkage.
5. The absorption band around 640–670 cm^−1^ has been observed for *ν*P=S vibrations and absorption band present in the region 550–580 cm^−1^ is due to *ν*P−S vibrations. Ring vibrations have been observed in the region 964–950 cm^−1^.

#### 2.1.2. ^1^H NMR spectra

^1^H NMR spectra of these derivatives are listed in Table 3. The spectra show the characteristic resonance for alkyl and glycoxy groups present on phosphorus. A multiplet at *δ* 4.7–4.9 ppm is assigned for glycoxy protons, which is due to long-range coupling of these protons with magnetically active phosphorus atom. A singlet appears for CH_2_ proton of succinimide ring at *δ* 1.1–1.5 ppm.

#### 2.1.3. ^31^P NMR spectra

In the proton decoupled ^31^P NMR spectra only one ^31^P NMR signal has been observed for each compound (see Table 3). Only one sharp resonance signal at *δ* 58–62 ppm shows purity of these compounds. An upfield shift (*δ* 19–22 ppm) is observed for these derivatives in comparison to parent dialkyl (alkylene) dithiophosphates, suggesting the covalent nature of newly formed sulfur-nitrogen linkage as well as the absence of any coordinating tendencies in the above derivatives. Thereby appears a unidentate nature of dithiophosphate moiety.

**Figure 2:**
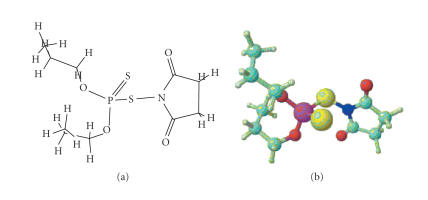
1-[(dipropoxyphosphorothioyl)thio] pyrrolidine-2,5-dione.

**Figure 3:**
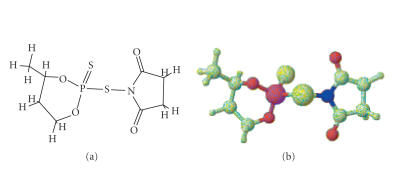
1-[(4-methyl-2-sulfido-1,3,2-dioxaphosphinan-2-yl)thio] pyrrolidine-2,5-dione.

On basis of the above spectral [IR, NMR (^1^H, ^31^P)] and other physicochemical evidences, the formation of [P−S−N] chemical bond has been established and the structures in Figures 2 and 3 have been tentatively assigned for the above derivatives.

#### 2.1.4. Experimental

Solvents were dried by standard methods. Ammonium salt of dialkyl/alkylene dithiophosphate was prepared by the methods reported in the literature [14]. Sulphur was estimated gravimetrically as barium sulphate (messenger method) [15]. Molecular weights were determined by the “Knaur Vapor pressure Osmometer” using a chloroform solution at 45°C. IR spectra were recorded in Nujol mull (4000–200 cm^−1^) on an FT IR spectrophotometer model Megna-IR-550 MICOLAC-USA. Carbon and hydrogen analyses were performed on a Perkin Elmer CHN/O analyzer. ^1^H NMR spectra were recorded in CDCl_3_ solution on a 90 MHz JEOL FX 90 spectrometer using TMS as an internal reference. ^31^P NMR was recorded in C_6_H_6_ using H_3_PO_4_ as an external reference (CDRI Lucknow). The experimental details of representative compounds are described below. Analytical results are summarized in Table 4.

#### 2.1.5. Preparation of $\overset{⎴}{{\text{OC(Me)}}_{\text{2}}{\text{CH}}_{\text{2}}\text{CHMeOP}}\text{(S)S}\overset{⎴}{{\text{NC(O)CH}}_{\text{2}}{\text{CH}}_{\text{2}}\text{C}}\text{(O)}$

An anhydrous acetonitrile solution (50 mL) of N-bromosuc-cinimide (0.98 g) was added into the suspension of ammonium salt of hexylene dithiophosphate (1.33 g) and refluxed for 6-7 h. Ammonium bromide precipitated within the course of reaction was filtered off and the solvent was removed under reduced pressure. A red/brown-colored sticky liquid product (1.8 g, 82.21%) was isolated. Synthetic and analytical data are given in Table 4.

## Figures and Tables

**Table 1:**
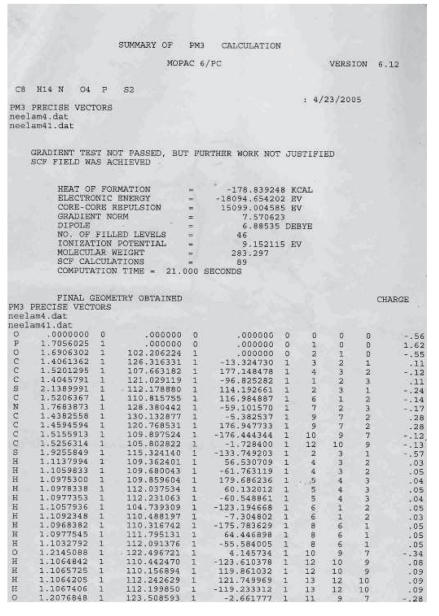


**Table 2:**
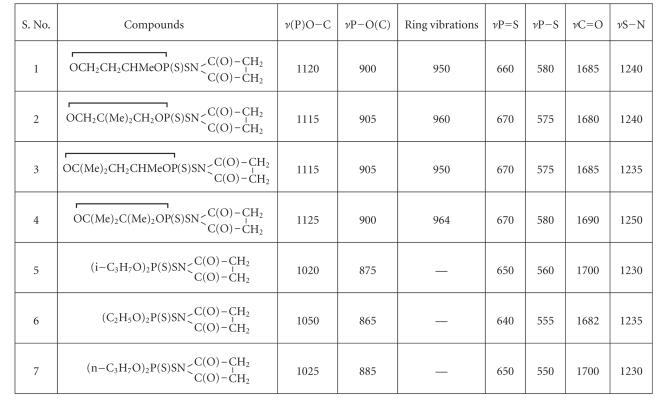
IR spectral data of N-bromosuccinimide derivatives of ammonium dialkyl/alkylene dithiophosphate.

**Table 3:**
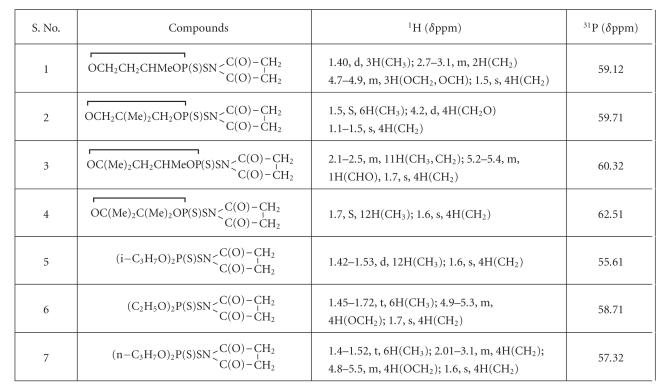
NMR ^1^H and ^31^P spectral data of N-bromosuccinimide derivative of ammonium dialkyl/alkylene dithiophosphate.

**Table 4:**
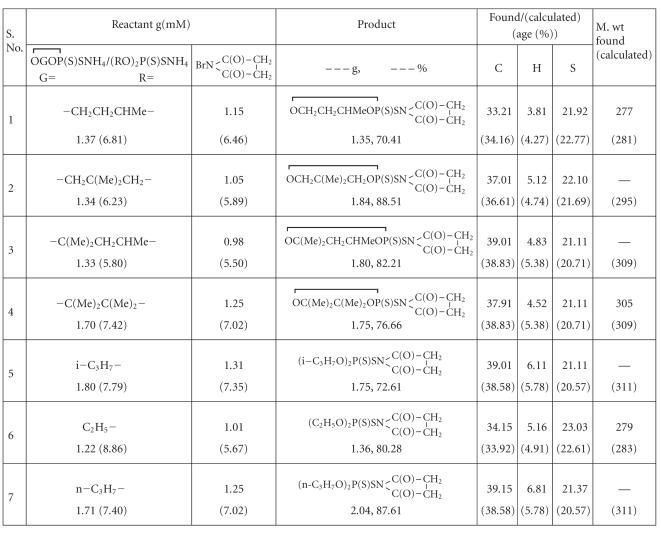
Synthetic and analytical data of N-bromosuccinimide derivatives of ammonium dialkyl/alkylene dithiophosphate.
